# Do alternative scaffolds used in regenerative endodontics promote better root development than that achieved with blood clots?

**DOI:** 10.1590/0103-6440202204746

**Published:** 2022-04-29

**Authors:** Letícia de Araújo, Taynara Santos Goulart, Ana Clara Kuerten Gil, Daniela Peressoni Vieira Schuldt, Beatriz Serrato Coelho, Daniela de Rossi Figueiredo, Lucas da Fonseca Roberti Garcia, Josiane de Almeida

**Affiliations:** 1 Department of Endodontics, University of Southern Santa Catarina (UNISUL), Palhoça, Santa Catarina, Brazil; 2 Department of Public Health, UNISUL, Palhoça, Santa Catarina, Brazil; 3 Department of Dentistry instead of Department of Endodontics, Federal University of Santa Catarina (UFSC), Florianópolis, Santa Catarina, Brazil

**Keywords:** Regenerative endodontics, tissue engineering, biocompatible materials

## Abstract

The aim of this integrative review was to identify whether alternative scaffolds used in regenerative endodontics contribute to better root development, in relation to the increase in root length and thickness of dentin walls, compared with blood clot (BC) scaffolds. The literature search was conducted in PubMed, SciELO and Lilacs databases, using descriptors related to the topic. After applying the eligibility criteria, 11 articles were selected and analyzed according to the proposed aim. Five clinical and six *in vivo* studies, conducted in animals, compared different types of alternative scaffolds with BCs, with emphasis on platelet-rich plasma (PRP) and platelet-rich fibrin (PRF). All scaffolds, alternative or BC, promoted an increase in root length and dentin wall thickness, with varying percentages of increase between studies. In general, there was a significant increase in root length and dentin thickness promoted by PRF and PRP scaffolds, compared with BC. It was concluded that the majority of the scaffolds tested contributed to the increase in root length and thickness of dentin walls, with emphasis on PRF and PRP.

## Resumo

O objetivo desta revisão integrativa foi identificar se os scaffolds alternativos utilizados em endodontia regenerativa contribuem para um melhor desenvolvimento radicular, em relação ao aumento do comprimento e espessura das paredes da dentina, em comparação com os scaffolds de coágulo sanguíneo (BC). A pesquisa bibliográfica foi realizada nas bases de dados PubMed, SciELO e Lilacs, utilizando descritores relacionados ao tema. Após a aplicação dos critérios de elegibilidade, 11 artigos foram selecionados e analisados de acordo com o objetivo proposto. Cinco estudos clínicos e seis in vivo, realizados em animais, compararam diferentes tipos de scaffolds alternativos com BCs, com ênfase no plasma rico em plaquetas (PRP) e fibrina rica em plaquetas (PRF). Todos os scaffolds, alternativos ou BC, promoveram um aumento no comprimento da raiz e na espessura da parede dentinária, com percentuais variáveis de aumento entre os estudos. Em geral, houve um aumento significativo do comprimento da raiz e da espessura da dentina promovido pelos scaffolds PRF e PRP, em comparação com a BC. Concluiu-se que a maioria dos scaffolds testados contribuiu para o aumento do comprimento das raízes e da espessura das paredes dentinárias, com ênfase em PRF e PRP.

## Introduction

Regenerative endodontic procedures (REPs) have been highlighted as a promising alternative to apexification, and like those such as revitalization or revascularization strategies ^1^, for example, they do not require periodic changes of medication, do not require the canal to be filled and, mainly, they allow the formation of a tissue rich in blood supply and progenitor cells, vital for the completion of rhizogenesis ^2^. However, despite the favorable clinical evidence ^3^, there are some limitations regarding the technique ^4^.

In the contemporary scenario of regenerative endodontics, in which the presence of stem cells, growth factors and a favorable environment for their development are necessary ^2^ *,* scaffolds have received great attention ^2^ ^,^ ^4^. Scaffolds are three-dimensional structures used inside the root canal, which provide a microenvironment capable of allowing the migration, proliferation, adhesion and differentiation of stem cells, as well as revascularization ^4^ ^,^ ^5^, with consequent thickening of the dentin walls and the conclusion of root development ^6^. In addition, they must reproduce the physical, chemical and biological characteristics of the pulp ^5^. At present, the majority of REPs are based on the use of endogenous or natural frameworks ^3^ ^,^ ^4^, such as a blood clot (BC) (7), platelet-rich plasma (PRP) ^8^ ^,^ ^9^ and platelet-rich fibrin (PRF) ^10^, which are favorable due to their cost, inflammatory, immune and toxicity response ^11^. However, they have technical limitations, such as difficulty in forming an intracanal clot after inducing bleeding or performing venipuncture to obtain PRF and PRP ^8^ ^,^ ^9^. PRP also has a short platelet life ^12^ and unlike PRF, it requires the addition of exogenous agents such as thrombin ^7^.

A wide variety of biomaterials, both natural and synthetic, are available for use as scaffolds ^13^, offering unique composition, structure, degradation profile and possibility of modification ^13^. Natural polymers, composed of hyaluronic acid and chitosan (HAC) ^14^, and pectin and chitosan (PC) ^14^, prioritize their chemical structure, are capable of mimicking the native tissue, and contributing to biocompatibility ^15^. Synthetic polymers, such as extrinsic matrix based on synthetic gelatin (SG) ^16^, extrinsic matrix based on synthetic fibrin (SF) ^16^, and injectable hydrogel scaffold impregnated with basic fibroblast growth factor (bFGF/FGF) ^17^, have the capacity for being reproducible, thus offer precise control of their mechanical and degradation capacities ^15^.

Despite the variety of potential biomaterials such as three-dimensional matrices, none of them has all the properties of an ideal framework, and the results related to the stimulation of root development are still varied and contradictory. Sometimes alternative scaffolds provide better results than BCs ^7^, sometimes they are equally effective and provide comparable results in terms of increasing root length and dentin wall thickness ^11^. Regarding to these clinical features, satisfactory outcomes based on the use of natural and derived from host scaffolds would increase their feasibility and bring not only the clinician closer to the regenerative procedures, but also the patient to an alternative treatment for their immature permanent teeth, strengthening the tooth against fracture ^18^ and improving its stability in the dental alveolus ^18^.

In view of the contradictions found in the scientific literature, the aim of this integrative review was to find out whether the alternative scaffolds used in REPs contribute to better root development when compared with BC scaffolds.

## Materials and methods

### Type of study

This integrative review was characterized as a qualitative, retrospective, documentary, and descriptive study. It was conducted in order to answer the following question: "Do alternative scaffolds used in regenerative endodontics contribute to better root development, in terms of increased root length and dentin wall thickness, than CS scaffolds?”. The PICO question was adjusted to the issue, as follows:

- P (Participants) - Alternative scaffolds
- I (Intervention) - Regenerative Endodontic Procedures
- C (Comparison or control) - Blood Clot
- (Outcome measure) - Root development

### Database

Individual search strategies were performed in the following electronic scientific databases: PubMed (https://pubmed.ncbi.nlm.nih.gov), Latin American and Caribbean Health Sciences Literature (Lilacs) (https://lilacs.bvsalud.org) and Scientific Electronic Library Online (SciELO) (https://www.scielo.org). All searches were conducted by March 18, 2021.

### Search strategy

**Chart 1:**
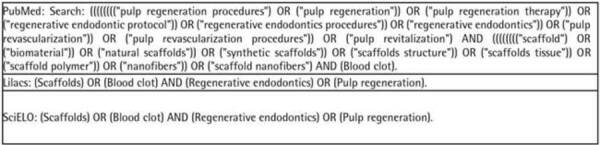
Set of keywords used in each database.

Appropriate keywords were selected to carry out this study. For each database, a combination of the following terms was used, as described in Chart 1, below:

### Eligibility Criteria

###  Inclusion criteria 

Studies that have compared alternative scaffolds with the BC scaffold in REPs; presence of descriptors; articles published in Portuguese, English and Spanish; articles published between 2010 and 2021; *in vivo* studies; clinical studies; studies that evaluated the increase in root length and/or thickness of dentin walls; without restriction as to the method of evaluation.

###  Exclusion criteria 

Studies were excluded based on the following criteria: articles with literature review only; articles with incomplete data; articles repeated between databases; articles with only abstracts available; letters and books; studies that evaluated other variables related to the pulp revascularization process.

### Selection of studies

Triage of articles was performed in two stages. In the first stage, the titles were read, and after this, the abstracts. In the second stage, the texts were read in full, and the articles that contemplated the inclusion criteria were selected. 

In Figure 1, a flow diagram is presented, containing the process of identification, inclusion and exclusion of the articles. The searches in the databases were performed up to March 18, 2021, and 15 articles were found. Three duplicated articles were removed, and a total of 49 studies were selected for analysis in Phase 1. After reading the title and abstract, 13 articles were selected for Phase 2. Based on reading the texts in full, 2 articles were excluded, totaling 11 articles that contemplated the eligibility criteria and were included in the integrative review.

**Figure:**
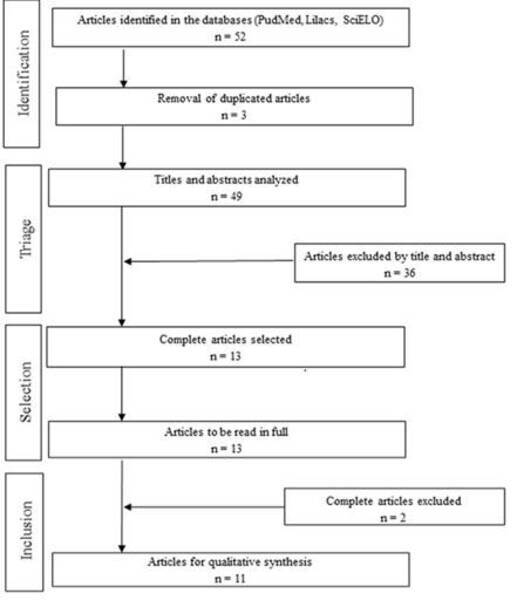
1. Flow Diagram of the search in databases, considering the selection criteria

### Data collection

For all articles included, the following descriptive characteristics were recorded: author, year, type of study, types of scaffolds, number of samples, properties evaluated (root length and/or dentin wall thickness), method of analysis, final experimental period and result, in relation to the increase in root length and/or thickness of the dentin wall. A researcher collected the data from the articles selected. A second researcher checked the information collected and confirmed its accuracy.

Clinical studies were submitted to the methodological quality analysis proposed by Jadad et al. ^19^, shown in Table 1, based on parameters such as randomization and study method, double-blind study, and description of exclusions or losses throughout the study. To fill in the table, the numbers “0” and “1” were used to determine the answers as “no” and “yes”, respectively. In the end, the sum of the answers generated a score, which determined the quality of the study, studies that totaled scores below 3 were considered to be of low quality, and studies that totaled scores above 4 were considered very good.

#### BC x PRF and PRP

**Table 1:** Methodological quality analysis of clinical studies, based on Jadad (1996) scale.

	Selected Studies				
	E1	E2	E3	E4	E5
Was the study described as randomized?	1	1	1	1	1
Was the study described as double-blind?	0	0	0	0	1
Was there a description of exclusions and losses?	1	1	1	1	1
Was the method used to generate the randomized sequence described and appropriate?	1	1	1	1	1
Was the double-blind method described and appropriate?	0	0	0	0	1
Score	3	3	3	3	5

0: NO / 1: YES / E1: Nagy et al. / E2: Alagl et al. / E3: Hongbing et al. / E4: Ulusoy et al. / E5: Rizk et al.

Animal studies were evaluated according to the SYRCLE ^20^ risk of bias scale (Table 2), which consists of 10 cue questions related to selection bias, performance, detection, attrition, reporting, and other biases. To complete the table, the letters “S” and “N” were used to determine responses such as “low risk of bias” and “high risk of bias”, respectively. When the risk of bias was uncertain; that is, the answer was not clear in the body of the article, an asterisk (*) was used to fill in the table.

**Table 2:** Quality assessment of *in vivo* studies, based on SYRCLE scale.

STUDY	Selection bias			Performance bias		Detection bias		Attrition bias	Report bias	Other sources of bias
	1	2	3	4	5	6	7	8	9	10
Torabinejad et al.	*	Y	*	Y	N	*	N	*	*	Y
Benítez et al.	Y	Y	*	Y	Y	*	Y	*	*	Y
Stambolsky et al.	Y	Y	*	Y	Y	*	Y	*	*	Y
Palma et al.	Y	Y	*	Y	Y	*	Y	Y	*	Y
Halaby et al.	*	*	*	*	*	*	*	*	*	Y
Jang et al.	Y	Y	*	Y	Y	*	Y	Y	*	Y

Y - Yes (low risk of bias) / N - No (high risk of bias) / *- uncertain (uncertain risk of bias) / 1-Was the allocation sequence adequately generated and applied? / 2-Were the groups similar at baseline or were they adjusted for confounders in the analysis? / 3- Was the allocation adequately concealed? / 4- Were the animals randomly housed during the experiment? / 5- Were the caregivers and/or investigators blinded from knowledge which intervention each animal received during the experiment? / 6- Were animals selected at random for outcome assessment? / 7- Was the outcome assessor blinded? / 8- Were incomplete outcome data adequately addressed? / 9- Are reports of the study free of selective outcome reporting? /10- Was the study apparently free of other problems that could result in high risk of bias?

## Results

Eleven studies published in the last 10 years were included in this integrative review, of which 5 were clinical studies and 6 in vivo studies. *All articles evaluated the use of alternative scaffolds in REPs compared with BC, in relation to increased root length and/or dentin wall thickness.*

### Study characteristics

Different types of scaffolds were compared with the blood clot (BC) scaffold. Among them, PRP ^21^ ^,^ ^22^, PRF ^21^ ^,^ ^23^ ^,^ ^24^, PP ^21^, and bFGF/FGF ^17^ were used in clinical studies. In the *in vivo* studies, in animals, PRP ^18^ ^,^ ^25^ ^,^ ^26^, PRF ^27^, SG ^16^, SF ^16^, HAC ^14^ and PC ^14^ were used.

Of the five clinical studies, four evaluated the increase in root length and dentin wall thickness, among other variables ^15^ ^,^ ^17^ ^,^ ^23^ ^,^ ^24^, by means of radiographic examinations. One study reported only the increase in root length ^22^, verified by cone beam computed tomography (CBCT) ^22^. Among the *in vivo* animal studies, four evaluated the increase in root length and dentin wall thickness ^14^ ^,^ ^16^ ^,^ ^18^ ^,^ ^27^, and two analyzed only the increase in wall thickness ^25^ ^,^ ^26^. The majority of studies used radiographic examination as the method of analysis, followed by histological analysis.

The results obtained by means of data analysis were presented quantitatively, as an increase in root length and dentin thickness in millimeters ^17^ ^,^ ^22^ ^,^ ^24^ ^,^ ^27^, as a percentage of increase ^24^ ^,^ ^27^, or as a percentage of cases in which there was or was no increase in the variables evaluated ^14^ ^,^ ^16^ ^,^ ^23^ ^,^ ^18^ ^,^ ^25^ ^,^ ^26^. The descriptive characteristics of the articles included can be seen in Table 1, considering the clinical studies, and in Table 2, of the *in vivo* studies.

### Methodological quality assessment

Based on the qualitative scale by Jadad et al. ^19^, all clinical studies described the randomization sequence that was shown to be appropriate for each investigation. Although all the studies evaluated had well-delineated methodology designs, only one described the method of analysis as being double blind. Therefore, among the articles included, four had 3/5 points on the Jadad et al ^19^ scale. The study by Rizk et al. ^24^, considered all of the evaluation criteria, generating the highest score (5/5).

When *in vivo* studies, conducted with animals, were qualitatively evaluated, the majority did not show situations that could generate a high risk of bias. Among the questions present in the SYRCLE scale, none of the studies could clearly answer the questions “Was the allocation adequately concealed?”, “Were animals selected at random for outcome assessment?” and “Are reports of the study free of selective outcome reporting?”, leaving these questions uncertain for risks of selection bias, detection and reporting bias, respectively, among the studies. Among the studies included in this review, only one showed high risk of bias relative to two of the questions ^18^, when addressing the issues of performance bias and detection bias, as they identified the types of intervention that the animals received during the experiment and during evaluation of results. Another study showed an uncertain risk of bias in 9/10 of the questions analyzed and were classified as a low-quality article ^27^. The remaining studies were considered to have a low risk of bias.

### Clinical Studies and type of scaffold

The results described as follows are with reference to the data shown in Box 1.

**Box 1:**
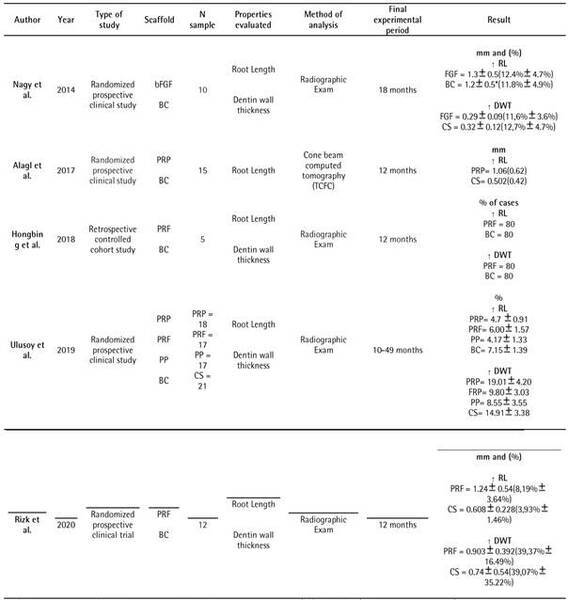
Descriptive characteristics of the clinical studies included.

bFGF Injectable hydrogel Scaffold impregnated with basic fibroblast growth factor / BC: Blood Clot / PRP: Platelet rich plasma / PRF: Platelet rich Fibrin/ PP: Platelet clumps / mm: millimeters / **↑:** Increase / RL: Root length/ DWT: Dentin wall thickness.

###  BC x PRF and PRP 

An increase in root length and thickness of dentin walls was observed, by means of radiographic examination, in the three clinical studies that evaluated the scaffolds of PRF and BCs ^21^ ^,^ ^23^ ^,^ ^24^. However, only one of them showed a significant increase in root length, promoted by PRF (8.19%), after a period of 12 months, compared with BC (3.93%) ^24^. Even after the 49-month follow-up period, no significant differences were observed between PRF and BC, both in relation to the increase in root length (PRF 6% / BC 7.15%) and in dentin thickness (PRF 9.80% / BC 14.91%) ^21^. When evaluating the percentage of cases, 80% of patients with PRF and BC had increased root length and dentin wall thickness after 12 months ^23^.

Two clinical studies compared PRP with BC ^21^ ^,^ ^22^. After a period of radiographic evaluation that ranged from 10 to 49 months, there was no significant difference between the PRP and the BC, for both variables analyzed ^21^. Although the value promoted by BC (7.15%) was higher than that of PRP (4.74%) for increasing root length, when the increase in dentin wall thickness was evaluated, a higher value was found for PRP (19.01%) compared with BC (14.91%) ^21^. However, when root length was assessed using CBCT, after 12 months, the PRP scaffold promoted a significant increase, approximately 0.5 mm more, compared with that promoted by BC ^22^. In addition, continuous root development was observed in 22 teeth (73% of cases), 14 with PRP and 8 with BC ^22^.

###  BC x other scaffolds 

Two other types of scaffolds were also evaluated, namely PP ^21^ and bFGF/FGF ^17^. For both variables analyzed, there was no significant difference in percentage between the scaffolds PP and BC, after 49 months ^21^. However, in cases in which BC was used, higher values of root length (BC 7.15% / PP 4.17%) and dentin wall thickness BC 14.91% / PP 8.55%) were observed ^21^.

There were no significant differences in mm and percentage of increase between FGF and BC scaffolds after 18 months of follow-up for both variables analyzed, with similar values found between groups. However, the analyses were performed in the time intervals of 3, 6, 12 and 18 months. When the final time interval of 18 months was compared with the other periods, for each scaffold, a significant difference was observed in the increase in dentin thickness for FGF and BC, and in root length for BC ^17^.

### *In vivo* studies and type of scaffold 

The results described as follows refer to the data shown in Box 2.

**Box 2:**
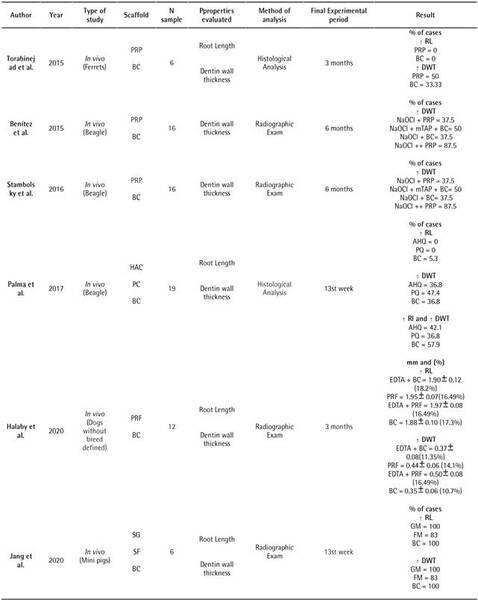
Descriptive characteristics of the *in vivo* studies included.

PRP Platelet rich plasma / BC: Blood Clot / HAC: Hyaluronic Acid and Chitosan / PC: Pectin and Chitosan / PRF: Platelet rich Fibrin/ SG: Synthetic gel-based extrinsic Matrix Synthetic fibrin-based extrinsic Matrix / mm: millimeters / **↑:** Increase / RL: Root length/ DWT: Dentin wall thickness / NaOCl: Sodium Hypochlorite/ mTAP: Triantibiotic Paste modified / EDTA: Ethylenediaminetetraacetic acid.

###  BC x PRP e PRF 

Histological analysis showed that when the PRF scaffold was used on ferret teeth for 3 months, it promoted an increase in the thickness of the dentin walls in 50% of cases, with no significant difference when compared with 33.33% of cases treated with BC ^18^. However, in procedures performed in Beagle dogs and analyzed by means of radiographs after 6 months, a significantly higher percentage of cases (87.5%) showed increased thickness of dentinal walls when PRF was used in combination with prior disinfection with sodium hypochlorite solution (NaOCl) and modified triantibiotic paste (mTAP), when compared with the use of NaOCl + BC solution (37.5%) and NaOCl + mTAP + BC solution (50%) ^25^ ^,^ ^26^.

Only one *in vivo* study, conducted with mixed breed dogs, radiographically evaluated the use of the PRF scaffold after a final period of 3 months ^27^. When the variable analyzed was the increase in dentin wall thickness after 3 months, a statistically significant difference was found for PRF (14.1%) and for PRF in conjunction with the prior use of ethylenediaminetetraacetic acid (EDTA) (14 .9%), when compared with the BC (10.7%) ^27^. When the variable analyzed was the increase in root length after 3 months, a statistically significant difference was found only for PRF + EDTA (18.9%), when compared with the BC (17.3%) ^27^. 

###  BC x other scaffolds 

Other types of scaffolds, namely SG ^16^, SF ^16^, HAC ^14^ and PC ^14^, were evaluated in other *in vivo* studies, in time intervals of 12 ^16^ and 13 ^14^ weeks, by means of radiographic ^16^ and histological analysis ^14^. A percentage of similar cases, in terms of increased root length and dentin wall thickness, was observed among SG (100%), SF (83%) and BC (100%) scaffolds, used in mini pigs and evaluated radiographically after 12 weeks ^16^. After 13 weeks, the HAC and PC scaffolds used in Beagle dogs, promoted an increase in the thickness of the dentinal walls that was histologically observed, in 36.8% and 47.4% of cases, respectively, similar to the increase promoted by BC (36.8%). Although there was no significant difference for the two variables analyzed, HAC and PC promoted no increase in root length, differing from the 5.3% of cases with BC, in which evidence of an increase was found ^14^.

## Discussion

Pulp regeneration/revascularization is a relatively recent procedure in the field of endodontics, and there is still no consensus about the ideal protocol to be followed. Many cases using different types of scaffolds have been reported in the literature ^14^ ^,^ ^16^ ^,^ ^17^ ^,^ ^18^ ^,^ ^19^ ^,^ ^20^ ^,^ ^21^ ^,^ ^22^ ^,^ ^23^ ^,^ ^24^ ^,^ ^25^. The most widely used scaffold and accepted at present is the type obtained by stimulating apical bleeding, with subsequent formation of the BC ^7^. This guided endodontic repair process allows for continuous root development, thickening of the root canal walls, apical closure and complete resolution of apical periodontitis ^28^. However, it is not always possible to obtain this bleeding, or the bleeding is frequently found to be insufficient ^21^, and with a limited the concentration of growth factors that are essential in REPs ^29^. Therefore, this integrative review investigated the evidence available in the literature regarding the alternative scaffolds used in REPs and whether they contributed to better root development when compared with the BC.

Among the various types of scaffolds analyzed, only PRF and PRP, both in clinical and *in vivo* studies, provided better results than BC ^21^ ^,^ ^22^ ^,^ ^23^ ^,^ ^27^. In clinical studies, after 12 months of follow-up, PRF ^24^ and PRP ^22^ promoted a significant increase in root length when compared with BCs. In line with these results, other studies have also shown that PRP and PRF ^22^ ^,^ ^30^ were more effective than BC in the process of root development. One of the components of these types of scaffolds are platelets, rich in cytokines and signaling molecules that play an essential role in cell differentiation ^24^. PRF is a bioactive molecule capable of creating a three-dimensional architecture and a suitable microenvironment for cell migration ^7^. Its use stimulates cell proliferation and increases the expression of specific proteins related to odontoblast differentiation ^7^. PRP stimulates collagen production, contains and releases many growth factors, and also retains and stimulates the proliferation of undifferentiated mesenchymal and endothelial cells found in the periapical region ^7^. However, paradoxically, no clinical study has shown evidence of a significant increase in dentin wall thickness provided by PRP and PRF, when compared with BC, even after long follow-up periods. PRF and PRP were only capable of increasing significantly longer root length than the BC ^21^ ^,^ ^22^ ^,^ ^24^ ^,^ ^27^. The difference in results could be justified by the different methods of evaluating the tissue and by the different protocols applied during performance of the treatment ^21^.

Whereas, in *in vivo* studies, PRF scaffolds, with prior irrigation with EDTA ^27^ and PRP ^25^ ^,^ ^26^ promoted a significant increase in dentin wall thickness and root length values ^27^ that were many times higher than the values obtained with BC, after time intervals of only 3 ^27^ and 6 ^25^ ^,^ ^26^ months. The action of surface demineralization of dentin and exposure of collagen fibers resulting from the use of EDTA, prior to the use of PRF scaffold, exposes part of the organic portion of the dentin matrix and its morphogenic proteins (growth factors), thereby contributing to root development ^31^. The EDTA irrigation protocol optimizes the environmental conditions for tissue regeneration, because in addition to allowing the survival of stem cells from the apical papilla, it also partially reverses the cytotoxic effects of NaOCl solutions, thus contributing to cell differentiation ^32^.

Clinical studies, in humans, and *in vivo*, in animals, have shown certain differences in their findings related to root development. Despite being an animal model closer to humans than that of rats ^33^, apical closure in ferrets occurred almost 2 months after tooth eruption, a shorter period of development than that observed in dogs and humans ^18^. Whereas the swine and dog, the animal model most used in the studies of this integrative review, showed similarities with humans with regard to the similarity of root structure, function ^30^ and apical repair ^30^. Nevertheless, these animals have disadvantages, such as their rapid development, which makes the results are achieved in experimental tests that occur in periods of short duration. The use of very young animals leads to results that cannot be compared with the adult human physiology ^33^.

In addition, the longer experimental periods of up to 49 months used in clinical studies ^21^, when compared with the maximum period of 6 months in *in vivo* studies, may have influenced the findings of the investigations, and allowed the BC to be as efficient in root development as the other types of scaffolds, including PRF and PRP. In a normal situation, the tooth can take up to 4 years to complete its root formation ^25^. Consequently, BC may need more time to play its full role as scaffolding in REPs. 

Despite the interesting results of the present integrative review, some limitations should be considered. Diverse methodologies were observed in the *in vivo* and clinical studies included, with differences regarding number of samples, method, and period of analysis to determine root development. Thus, the comparison among studies should be carefully interpretated and may lead to a restricted conclusion. Also, it is worth mentioning that in the *in vivo* studies there was a variation among the type of animal used, such as dog ^14^ ^,^ ^25^ ^,^ ^26^ ^,^ ^27^, ferret ^18^, and mini pig ^16^, what may reflect in different results, due to the different biological responses. Yet, in this integrative review, most of included clinical and *in vivo* studies used radiographic exam to determine root development ^16^ ^,^ ^17^ ^,^ ^21^ ^,^ ^22^ ^,^ ^23^ ^,^ ^24^ ^,^ ^25^ ^,^ ^26^ ^,^ ^27^. Radiographs provide a two-dimensional (2D) image of tridimensional (3D) objects, which can render a distorted anatomic image of the tooth or overlap adjacent structures ^34^. Due to these drawbacks, cone-beam computed tomography (CBCT) 3D image became an essential tool in endodontics, especially to evaluate REPs outcomes ^34^. Of the evaluated studies, only one used this feature for root development analysis, expressing possibly more accurate results ^22^.

In addition to CBCT, it is worth mentioning the importance of the pulp vitality tests to identify REP success. Currently, most of the studies about regenerative endodontics evaluated repair issues, such as root development, rather than regenerative issues, such as the observed with pulp vitality ^35^. Some authors ^36^ ^,^ ^37^ reported the growth of a vital tissue inside of the root canal capable of responding to thermal (cold) and electric vitality tests in 50% of the cases ^37^. However, histological information about the type of tissues formed in the root canal space and the vasculogenesis and neurogenesis process is still scarce ^35^.

Although numerous requirements must be considered when selecting an appropriate scaffold to support stem cells, such as biocompatibility, architecture, mechanical strength, and biodegradability ^16^, the present integrative review showed that all the scaffold analyzed were clinically effective and functional, promoting further root development and consistently result in the formation of new calcified tissue to increase both root thickness and length. Thus, strengthening the tooth against fracture ^18^ and improvement of its stability in the dental alveolus ^18^ is expected. Once disinfection has been carried out, the “predictable” clinical outcomes associated with REPs when natural and derived from host scaffolds are used, such as blood clot, PRP and FRP could increase their feasibility and bring the clinician closer to this promising alternative treatment for immature permanent teeth, which promotes healing of affected tissues, as well as patient welfare ^37^.

Based on the findings of this integrative review, it was noted that the majority of alternative scaffolds showed results that were very similar to those of BCs in terms of stimulating root development, with only PRF and PRP being outstanding. It is, therefore, possible for these scaffolds to become a feasible alternative for the treatment of teeth with incomplete rhizogenesis, given their potential to release growth factors, and their ability to stimulate and initiate tissue repair ^11^. However, as it is a relatively new treatment, little is known about its long-term effects ^7^, which requires caution and shows the need for further clinical and laboratory research, in order to establish an ideal protocol for REPs.

## Conclusion

The present integrative review showed that all scaffolds, alternative or BC type, promoted an increase in root length and dentin wall thickness, with emphasis on the alternative PRF and PRP scaffolds.
